# Reading and Writing Development in Inclusive Settings: Teachers’ Perception of the Use of Digital Technology

**DOI:** 10.3390/bs15050682

**Published:** 2025-05-16

**Authors:** Lénia Carvalhais, Paula Vagos, Lídia Ferreira Cerejeira, Teresa Limpo

**Affiliations:** 1 Department of Psychology and Education, University Portucalense, 4200-072 Porto, Portugal; 2 Life Quality Research Centre (CIEQV), 2001-964 Santarém, Portugal; 3 William James Center for Research, University of Aveiro, 3810-193 Aveiro, Portugal; paulavagos@ua.pt; 4 Escola Superior de Educação, Instituto Politécnico de Santarém, 2001-904 Santarém, Portugal; lidiaifc@gmail.com; 5 Faculty of Psychology and Education Sciences, University of Porto, 4200-135 Porto, Portugal; tlimpo@fpce.up.pt

**Keywords:** reading, writing, inclusion, teachers, digital technology

## Abstract

Reading and writing are foundational skills throughout school grades that could be improved using digital technology, especially in inclusive contexts. The present study aimed to understand primary teachers’ use of technology and their self-efficacy perception in the use of digital technology in inclusive settings, considering their application of universal, selective, or additional measures. In total, 290 Portuguese primary school teachers (1st to 4th grades) aged 25 to 66 years old (M = 49.32, SD = 8.59), mostly female, filled in an online survey, comprising a sociodemographic sheet and four questionnaires related to digital technologies. The results show that most teachers apply measures within the inclusive education framework (n = 277). The sociodemographic and educational profiles of teachers who use or do not use those technologies were similar, as were teachers’ self-efficacy scores about using digital technologies, perception about the utility of those technologies, and use of them, overall and for reading and writing specifically. The more teachers consider themselves efficacious in using digital technologies and the more they hold a positive perception of those technologies, the more they report their use. These findings reinforce the influence of teachers’ internal factors, such as self-efficacy, on the use of digital technologies, independent of students’ specific difficulties.

## 1. Introduction

Reading and writing are foundational skills that are critical for early childhood education, and their effective teaching is particularly relevant in inclusive settings (Fälth & Selenius, 2022). In inclusive classrooms, as advocated by the Salamanca Statement and Framework for Action on Special Education Needs (UNESCO, 1994), teachers are asked to ensure that all students, regardless of their diverse educational needs, have access to effective instruction. This effective instruction implies the practice of reading and writing abilities, including letter knowledge, phonological awareness, and writing or reading comprehension (Hutchison & Woodward, 2014). Over the last several decades, research has focused on understanding how digital technology can enhance reading and writing skills by providing differential teaching adapted to each student’s individual needs (Biancarosa & Griffiths, 2012; Fälth & Selenius, 2022; Lyytinen et al., 2009). Previous studies on using computer-assisted interventions with primary school students have highlighted these tools’ potential to bridge the literacy development gap among primary-grade students (Carvalhais et al., 2020). Computer-based teaching tools could be used in learning settings to promote individualized learning, with tasks being adapted to each child’s learning progress to provide responsive and immediate feedback on exercises (Lyytinen et al., 2007) and to potentially increase the student’s motivation (Richardson & Lyytinen, 2014), especially for those at risk for reading and writing difficulties (Blok et al., 2002; Nicolson et al., 2000). Nevertheless, teachers’ use of digital technology is still a challenge (Fernández-Batanero et al., 2020), as recent studies have shown that teachers in the European Union still report this use as a complex process, especially those who teach in the initial school years (Carvalhais & Azevedo, 2024; Pöntinen & Räty-Záborszky, 2020). Furthermore, during the initial training of future teachers (Janeš et al., 2023), and during their lifelong learning (Garzón Artacho et al., 2020), the development of digital competences continues to be a challenge, even with the advances of society in recent years (Santilli et al., 2025). Using digital technology in reading and writing instruction, specifically, can seem time-consuming for teachers (Christ et al., 2018), and constraints related to the equipment or access to the internet could hinder the daily use of technology in classrooms. When it comes to inclusive settings, the use of such technologies may be further hindered by additional challenges posed by these contexts (e.g., insufficient facilities for learning, difficulties in student-to-student interactions, lack of professional development opportunities and career valorization for teachers, or inadequate parent participation; Jardinez & Natividad, 2024).

In inclusive settings, implementing the Universal Design of Learning principles (for more information, see CAST, 2024; Pisha & Coyne, 2001), defined in Portuguese Decree-Law No. 54/2018 (Presidência do Conselho de Ministros, 2018), can promote literacy in a more flexible learning environment. These inclusive contexts accommodate the diversity of learning abilities in reading and writing, anticipating that children arrive at schools with wide differences in literacy abilities (Johler & Krumsvik, 2022). This paradigm of Universal Design of Learning is based on teaching, learning, curriculum, and assessment adaptations to a broader range of learners, including those with or without special needs, based on a multilevel approach to the curriculum (Decree-Law No. 54/2018, July 6) designed to ensure that all reach the expected student profile at the end of compulsory schooling, even if it is through differentiated learning paths. According to the above-mentioned law, measures to support learning and inclusion are implemented by schools and are divided into three intervention levels—universal, selective, and additional—mobilized according to each student’s educational needs and carried out by the teachers after the parent or guardian’s consultation. The universal measures correspond to more generalized procedures applicable to all students, including differentiated instructions, curricular accommodations, and enrichment (Decree-Law No. 54/2018, July 6). Selective measures are applied to fill the learning needs that are not met by universal measures and are dependent on the school’s material and human resources, including tutorial supportand differentiated curricular pathways. Finally, additional measures are oriented to students with “persistent communication, interaction, cognitive, or learning difficulties that require specialized resources and support to learning and instruction” (English version of Decree-Law No. 54/2018, July 6, p. 9). Additional measures include significant curricular adaptations and the development of structures, teaching methodologies, and strategies to support students, including the support of a special education teacher responsible for the differentiated learning process. According to Alves et al. (2020), who used data from the Portuguese General Directorate of Statistics of Education and Science (DGEEC) in 2018, 98.9% of students with special education needs in Portugal are included in mainstream schools comprising public and private contexts, which results in teachers facing several challenges in pedagogical differentiation and in supporting and supervising the implementation of these measures. In this context, materials and methodologies based on digital technology could leverage the learning process among students with some educational-specific needs.

Still, research on how teachers use technology in primary school grades to promote reading and writing skills is limited (Fälth & Selenius, 2022). On the one hand, it is crucial to understand how teachers perceive the usefulness of digital technology in terms of frequency of use, overall or specifically to teach reading and writing in inclusive settings. On the other hand, previous studies on the relevance of teachers’ self-efficacy reported that teachers with a strong self-efficacy about their ability to use a specific teaching tool are more likely to implement it in the classroom (Buabeng-Andoh, 2012; Nunes et al., 2022). Also, a study conducted in Swedish primary schools revealed that 74% of 289 primary school teachers used digital technology to support students with special needs every week, with those who reported perceiving digital technology as a facilitator of all students’ participation being the ones to promote different reading and writing skills more frequently through the use of digital technology (Fälth & Selenius, 2022).

Age, years of service, sex, and school years taught are sociodemographic and educational variables that have also been previously related to digital technology use in educational settings (Cantú-Ballesteros et al., 2017). Comparing younger and less-experienced teachers with older teachers, studies reported that older teachers were less familiar with and used less ICT (Information and Communication Technology) in their classrooms (Prensky, 2001). Considering different school grades, studies reported differences in the teachers’ digital proficiency (Carvalhais & Azevedo, 2024; Pöntinen & Räty-Záborszky, 2020). However, studies have focused on comparing teachers in primary and secondary school grades. Less is known considering primary teachers, in particular, who are responsible for initiating students into the formal learning of reading and writing. Female teachers were also less confident than male teachers in the use of ICT (Zhou & Xu, 2007). However, these results were not observed in other studies, with no differences being observed in the digital competence perceived by teachers according to age and years of service (Wang et al., 2012). In a study conducted by the International Computer and Information Literacy Study (ICILS) (Fraillon et al., 2019; Fraillon et al., 2025), teachers in lower secondary education (8th grade) were surveyed on the use of ICT in teaching, their confidence and self-efficacy in digital tasks, their attitudes toward ICT integration, and their professional development using ICT, through standardized instruments across all participating countries. The results from ICILS 2018 (Fraillon et al., 2019) pertaining to Portuguese teachers highlighted their moderate confidence in the use of ICT and reinforced the relevance of the use of ICT by students. Still, gaps in access to professional development using ICT were observed given that just a minority of teachers felt well prepared to teach using digital technology, in line with the results obtained from teachers in several other countries participating in this international study. Furthermore, the international results from the ICLS 2023 (Fraillon et al., 2025) reinforce the teachers’ needs for further professional development to incorporate digital technologies into their teaching practices, specifically relating to the more advanced digital resources.

This study has three main objectives. Firstly, we aim to characterize teachers based on their professional profiles and their use of digital technologies in the three tiers of inclusive multilevel interventions, from universal to selective and then to additional measures, while also comparing teachers’ self-efficacy and frequency of use of digital technology based on the three tiers of inclusive multilevel interventions. Secondly, we aim to understand teachers’ use of digital technologies to promote reading and writing skills, considering the relevance of sociodemographic, professional, and digital technologies-related profiles and the reported efficacy and perception of digital technologies. Thirdly, we aim to explicitly describe teachers’ use of digital technologies to promote reading and writing. Given our research goals, our hypotheses were as follows:

(1) There are no significant differences in the sociodemographic and training profiles, nor in their self-efficacy, perception of utility, and frequency of use of digital technologies reported by teachers using diverse levels of inclusive education measures.
(2) High perceptions of the ability to use technology and the perception of technology as a facilitator in teaching will lead to a high frequency of their use, independent of the inclusive context and measure implemented.
(3) Teachers frequently use digital technology to promote reading and writing skills.

## 2. Materials and Methods

### 2.1. Participants

The participants were 290 Portuguese primary school teachers aged 25 to 66 (M = 49.32, SD = 8.59) who had been teaching from less than 1 year to 45 years (M = 23.40, SD = 10.83). Most participants lived in the central region of Portugal, were female (89.3%), had a licensure degree (84%), had no special education specialization (89.3%), and taught in a public school (97.2%; see Table 1). All teachers taught at the primary school level, and many teachers taught more than one school year in the same class. Most participants self-reported that they were familiar with digital technologies (95.5%) and had had previous training in using them for educational tasks (72.4%).

### 2.2. Measures

All measures were used in their Portuguese version. Portuguese versions of the “Scale of Frequency in the Use of Technology”, “Scale—Promotion of reading and writing”, and “Scale—Teacher’s Perceptions of Digital Technology in Early Reading and Writing Education” were adapted and translated by the research team and will be made available upon a request directed to the corresponding author without undue reservation. The above-mentioned scales were translated following a translation process from English to Portuguese by two researchers, specialists in the topic and scales’ translation, a synthesis of translation by the research team, back-translation by a professional translator, and an analysis by a panel of teachers working in primary school grades.

#### 2.2.1. Self-Efficacy in the Use of ICT in the Classroom (Based on Nunes et al., 2022)

Following previous work conducted by Blackwell et al. (2014, 2016), teachers were presented with nine different ICTs that could be used in the classrooms for instructional purposes (viz., TV/DVD, laptop/desktop computer, digital camera or video recorder, interactive whiteboard, smartphone, e-reader, tablet, and assistive technologies). In the study of Nunes et al. (2022), questions were designed based on the frequency of use: for each ICT, respondents were asked to indicate how often they used it for instructional purposes, with a scale ranging from 0 (never) to 6 (daily). In the present study, the same items were presented using a scale of confidence from not at all confident (1) to very confident (5) in the use of the technology mentioned above. The scale had a very good internal consistency using the current sample: α = 0.80.

#### 2.2.2. Scale of Frequency in the Use of Technology (Adapted and Translated from Fälth & Selenius, n.d.)

Participants were asked to report the frequency of technology use, considering overall tasks (e.g., “For instruction and lectures for the whole class.”; Portuguese version: “Para dar instruções ou apresentar conteúdos à turma toda.”) and specific tasks related to reading and writing applied in special needs contexts (e.g., “As an extra adaptation in reading and writing education with students with additional measures; Portuguese version: “Como adaptação extra para o ensino da leitura e escrita, com alunos(as) abrangidos com medidas adicionais.”). The nine items were responded to using a scale that ranged from never (scored as 0), 1–2 times per semester (scored as 1), 1–2 times per month (scored as 2), every week (scored as 3), and several times a week (scored as 4). The research team translated and adapted the items from an initial scale by Fälth and Selenius, which comprises eight items. Due to the specificities of the Portuguese educational system, a new item was included to distinguish the application of exceptional support for students with universal and selective measures. The scale’s internal consistency was very good using the current sample: α = 0.84.

#### 2.2.3. Scale—Promotion of Reading and Writing (Translated to the Portuguese Language from Fälth & Selenius, 2022)

Participants were asked to identify how often they used digital technology to work on 14 different reading and writing skills during the last semester in their classes. The scale comprises 14 items: phoneme and grapheme knowledge, phonological awareness, decoding, reading fluency, reading comprehension, vocabulary, grammar, spelling, formulating thoughts or opinions, editing text, information searches, presentation of information, and listening comprehension (e.g., item 1: “Phoneme and grapheme knowledge”; Portuguese version: “Conhecimento grafema-fonema”). Participants reported the frequency of use of digital technology to develop students’ reading and writing from never (scored 0), 1–2 times per semester (scored 1), 1–2 times per month (scored 2), every week (scored 3), and several times a week (scored 4). The scale’s internal consistency was very good: α = 0.91.

#### 2.2.4. Scale—Teacher’s Perceptions of Digital Technology in Early Reading and Writing Education (Translated to the Portuguese Language from Fälth & Selenius, 2022)

The Scale—Teacher’s Perceptions of Digital Technology in Early Reading and Writing Education was translated by the research team into Portuguese, following a similar translation process of previously reported scales from Fälth and Selenius. Participants were asked to respond to 14 statements about their perception of digital technology in reading and writing education. A four-point Likert scale was presented: strongly disagree, disagree, agree, and strongly agree; the scores were 0 to 3. There were seven positive items and seven negative items, namely seven statements about how digital technology can be a facilitator of students’ participation in reading and writing tasks (e.g., “Digital technology benefits the reading and writing development of students who have reading and writing difficulties.”; Portuguese version: “A tecnologia digital beneficia o desenvolvimento da leitura e da escrita dos alunos com dificuldades nestas competências.”) and seven items about how technology could hinder the management of reading and writing activities in classes (e.g., “Extra planning time is required to use digital technology in reading and writing education.”; Portuguese version: “É preciso tempo de planeamento adicional para usar tecnologia digital no ensino da leitura e da escrita.”). In the original study, the value of Cronbach’s alpha for teachers’ perceptions of digital technology as a facilitator was α = 0.90, and the value for the second component of managing digital technology was α = 0.86. A reliability analysis was performed for the present study, and the value of Cronbach’s alpha for the positive subscale was α = 0.90 and for the negative α = 0.79.

### 2.3. Procedures

Data were collected online using the LimeSurvey software (version 5.3.17). The link to participate was disseminated through the email of directors of Portuguese schools/school groups, including schools from several national regions, who were asked to disseminate the study among primary school teachers. The link to participate in the study was made available during the second semester of the academic year of 2023/2024. Participants were asked to provide informed consent and then to respond to sociodemographic questions and the four questionnaires related to the use of technology in reading and writing in inclusive settings described above. The teachers’ participation in the present study was anonymous and voluntary, and teachers were asked to consent when submitting the online questionnaire.

This study was conducted with the approval of the translated versions’ authors, who permitted their use within the current research. The ethical procedures adhered to the Helsinki Declaration and national legislation reporting to the General Data Protection Regulation. The Ethical Committee of the Faculty of Psychology and Education Sciences of the University of Porto also approved the study (Reference 2024-05-02).

#### Data Analysis

Data analyses were carried out for each of this study’s goals. Descriptive analyses were considered for the first and third goals: to characterize teachers’ profiles based on the three tiers of inclusive multilevel interventions and to describe teachers’ use of digital technologies to promote reading and writing skills. Mean-group comparisons were also carried out within the first goal of this work to investigate differences in teachers’ reported self-efficacy, use of digital technologies, and perception of those technologies. Then, correlation and hierarchical regression analyses were used in relation to our second goal. The first was used to explore associations between teachers’ self-efficacy in using digital technologies and their perception of those technologies. The latter was used to investigate the relative importance of several aspects to the frequency of use of digital technologies, overall and related to reading and writing. Specifically, sociodemographic variables (i.e., sex, age, and geographical area) were entered in Step 1, variables relating to educational profile (i.e., education, specialization in special education, school type, school year taught, years of service, and special needs measures adopted) were entered in Step 2, variables related to digital technology profile (i.e., self-reported familiarity and previous training) were entered in Step 3, and self-efficacy and the perception of digital technologies were entered in Step 4.

## 3. Results

### 3.1. Teachers’ Characterization Concerning Inclusive Educational Measures

Only 13 teachers reported that their class did not include students with specific learning measures (n = 13, 4.5%). In contrast, most teachers taught classes where at least one specific learning measure was applied (n = 277, 95.5%). Specifically, 72 teachers applied only universal measures (24.8%), 20 teachers applied both universal and additional measures (6.9%), 114 teachers applied both universal and selective measures (39.3%), 4 teachers applied both additional and selective measures (1.4%), and 67 teachers applied universal, additional, and selective measures (23.1%).

For characterization purposes and considering the limited number of teachers who applied additional and selective measures, we considered teachers based on three groups: those who applied only universal measures, those who applied two types of measures, and those who applied all measures. Table 2 shows teachers’ sociodemographic and educational profile in relation to inclusive educational measures.

### 3.2. Use of Digital Technologies in Relation to Sociodemographic, Educational, and Technological Variables

There were no statistically significant differences in teachers who applied only universal measures, two types of measures, and all measures concerning self-efficacy in using digital technologies, the frequency of using digital technologies overall, and the frequency of using those technologies for teaching reading and writing in specific, nor in the perception of the use of digital technologies. The descriptive and correlation values between all these measures in the complete sample are presented in Table 3. Positive and significant correlation values were found between all measures, except for the negative perception of digital technologies that correlated negatively and significantly with all other measures (see Table 3).

Hierarchical regression analyses were then used to investigate the relative importance of demographic, professional, and technology-related variables, as well as self-efficacy and the perception of that technology, in relation to the frequency of use of digital technologies, overall and in reading and writing.

For the use of digital technologies overall, models considering Steps 1 through 3 were not statistically significant in explaining their use. The model considering all independent variables (i.e., Step 4) was statistically significant [*F*_(14,275)_ = 5.61, *p* < 0.300] and explained 23% of the variance of the self-reported use of digital technologies. Within that model, only self-efficacy and a positive perception of digital technologies were significant contributors to teachers’ use of digital technologies overall (see Table 4).

As for the use of digital technologies for reading and writing, two models were statistically significant: the model including Steps 1 through 3 [*F*_(11,275)_ = 2.16, *p* = 0.02; *r*^2^ = 0.08] and the model including all four steps [*F*_(14,275)_ = 4.87, *p* < 0.001, *r*^2^ = 0.21]. Also, the additional variance explained by adding Step 3 to Step 2 was statistically significant (Δ*r*^2^ = 0.02, *p* = 0.04) as was the added variance explained from Step 3 to Step 4 (Δ*r*^2^ = 0.12, *p* < 0.001). Still, considering all variables taken together (i.e., Step 4), self-reported familiarity with digital technologies and previous training in using them (added in Step 3) were not significant predictors of using such technologies in reading and writing; alternatively, self-efficacy and a positive perception of digital technologies were the only significant predictive variables in the analyses (see Table 4).

### 3.3. Use of Digital Technology to Promote Students’ Reading and Writing Skills

Most teachers used digital technologies to promote reading and writing skills weekly (more than 38.3%), mainly for supporting reading comprehension, vocabulary, and grammar (over 50% of the sample; Table 5).

## 4. Discussion

The use of technology for reading and writing in primary school grades is a relevant topic of research (Iinuma, 2016), assuming the complexity of implementing several educational measures that promote flexible learning environments. In the present study, we first aimed to characterize teachers based on their professional profile and use of technologies in the three tiers of inclusion, from universal to additional measures. We also compare teacher’s self-efficacy and frequency of use based on these three tiers of inclusive multilevel interventions. Furthermore, we hypothesized that sociodemographic variables, such as sex, age, and geographic area and the educational profile of teachers (i.e., education level, specialization in special education, school type, school year taught, or years of service) would not explain the frequency of use of technology for reading and writing activities and for general activities in the classroom. The results of the present study confirm our hypothesis and align with previous studies that did not identify these variables as significant in explaining the frequency of use of technology. Fälth and Selenius (2022) reported that teachers’ age was not significantly correlated with the frequency of promoting reading and writing skills. In the study by Nunes et al. (2022), similar results were obtained for gender and teaching experience, which did not explain the actual use of technology in classrooms. Secondly, we hypothesized that self-efficacy in using digital technology and the perception of technology as a facilitator in teaching would result in a high frequency of their use, independent of an inclusive context. Overall, we found that teachers’ self-efficacy in using digital technology and a positive perception of digital technologies in educational settings were significant in explaining the frequency of using digital technologies for reading- and writing-specific activities and for general activities in inclusive classrooms. These findings align with previous research reporting that the frequency of technology use depends on teachers’ self-efficacy to achieve this goal (Buabeng-Andoh, 2012; Nunes et al., 2022). Russell and Bradley (1997) mentioned that teachers’ personal characteristics, such as a lack of confidence and competence, often imply a reduced adoption of digital technologies in educational contexts. On the other hand, teachers who believe they can use digital technology reported more use in the classroom, independent of the measures for inclusive education implemented in their classrooms. Instead, using technology in reading and writing activities and overall teaching activities only focused on assistive technology to students with disabilities has been considered as labelling and discriminating (Johnston & Evans, 2005) and, in the end, could result in school abandonment (Van Laarhoven et al., 2012). From this perspective, digital technology should be used in inclusive classes that include children with several different learning measures to develop their reading and writing. In this sense, teachers’ frequency of using those technologies in the classroom should and seems to rely more on internal variables, such as the confidence to use different technology in classrooms, rather than on the specific knowledge obtained in this area. Furthermore, the positive perception of technology for reading and writing activities as a facilitator of all students’ participation in inclusive settings also predicted the frequency of use, which aligns with previous results in this field (Fälth & Selenius, 2022). Oppositely, teachers who negatively perceive technology or do not see it as a facilitator and found difficulties in managing digital technology in early reading and writing also reported less use of ICT. In a Cabero-Almenara et al. (2020) study, teachers who reported fewer digital skills also implemented less technology in teaching contexts. According to Nunes et al. (2022), the lack of technical support or access to equipment negatively predicted the effective use of technology. If these resources are lacking, teachers seem to prefer to use more traditional teaching methods. However, in our work, this negative perception was not a primary (significant) motive explaining the variance in their reported use of technology, overall and in tasks that involve reading and writing, such as phonological awareness, reading comprehension, and activities that imply presenting content.

Lastly, we hypothesized that teachers frequently use digital technology to promote reading and writing skills in their classes. Our results indicate that teachers report weekly use of technology during the last semester, mainly supporting reading comprehension, vocabulary, and grammar, indicating a high frequency of their practice. However, some teachers mention that they never use technology, mainly on tasks related to phoneme–grapheme knowledge or to formulate thoughts or opinions. In the study of Fälth and Selenius (2022), formulating thoughts or opinions was also one of the tasks most referenced by teachers as never taught through technology. In this sense, even if teachers seem familiar with the use of technology in reading and writing tasks, the complexity and specificity of each task may impact on the frequency of their implementation.

The current findings should be considered in light of some limitations. First, our sample was composed of teachers pre-disposed to take part in research initiatives and was imbalanced in relation to several demographic variables (e.g., sex or previous educational level). Though this may adequately characterize Portuguese teachers (i.e., our sample constitution is similar, for example, to that reported by Nunes et al., 2022), it nevertheless implies that our findings may apply particularly to the individuals mostly represented in our sample. Secondly, as a cross-sectional study, causality inferences should be avoided, with additional longitudinal studies being needed. Thirdly, the data were based on self-reports that could be influenced by social desirability processes and, hence, may not accurately represent teachers’ practices overall. Furthermore, data were collected through a new instrument used in a Swedish study and translated into Portuguese, with more studies needed to test the scales’ robustness and their ability to address the constructs under analyses in a cross-cultural framework, which would allow potential cultural and contextual-based differences among teachers working in different countries to be addressed.

## 5. Conclusions

Self-efficacy and the perception of technology as a facilitator for teaching reading and writing skills in inclusive classrooms explained the frequency of their use by teachers. Teachers seem to implement technology for all students, independent of their potential difficulties, resulting in the use of technology in a more inclusive rather than discriminatory support for teaching and learning. Furthermore, working on internal factors such as self-efficacy is crucial for promoting teachers’ confidence in implementing technology in their classes and their perception as a facilitator of students’ learning, overall and in reading and writing. These results may be particularly relevant for a deeper analysis and research on teacher-training programs for future primary school teachers, not only reflecting the knowledge about the use of digital technology, but also future teachers’ self-efficacy beliefs in their use. The results also showed that, depending on the different skills promoted, teachers seem to use technology diversely, thus reinforcing the idea that the complexity of tasks, for example, the formulation of thoughts or opinions, demands teachers’ abilities and flexibility in redefining their practices.

## Figures and Tables

**Table 1:** Sample characterization based on sociodemographic, educational, and background in familiarity with digital technologies and training.

		n	%
Sex			
	Female participants	259	89.3
	Male participants	31	10.7
Geographical area			
	North	86	29.7
	Center	139	47.9
	South	28	9.7
	Azores island	25	8.6
	Madeira island	12	4.1
Education			
	Licensure	244	84.1
	Master’s degree	42	14.5
	Doctorate	4	1.4
Specialization in Special Education			
	No	259	89.3
	Yes	31	10.7
School type			
	Public	282	97.2
	Private	8	2.8
School year taught			
	1st year	51	17.6
	2nd year	51	17.6
	3rd year	51	17.6
	4th year	57	19.7
	More than one year	80	27.6
Self-reported familiarity with digital technologies			
	No	13	4.5
	Yes	277	95.5
Training in digital technologies			
	No	80	27.6
	Yes	210	72.4

**Table 2:** Teachers’ characterization by categories of using measures within an inclusive education framework.

		Universal Measures (n = 72)	Two Measures (n = 138)	All Measures (n = 67)
Sex				
	Female participants [1]	64 (88.9)	126 (91.3)	58 (89.6)
	Male participants [2]	8 (11.1)	12 (8.7)	9 (13.4)
Geographical area				
	North [1]	23 (31.9)	46 (33.3)	15 (22.4)
	Center [2]	32 (44.4)	65 (47.1)	36 (53.7)
	South [3]	4 (5.6)	16 (11.6)	6 (9.0)
	Azores island [4]	10 (13.9)	7 (5.1)	7 (10.4)
	Madeira island [5]	3 (4.2)	4 (2.9)	3 (4.5)
Education				
	Licensure [1]	60 (83.3)	117 (84.8)	57 (85.1)
	Master’s degree [2]	12 (16.7)	18 (13.0)	9 (13.4)
	Doctorate [3]	0 (0.0)	3 (2.2)	1 (1.5)
Specialization in Special Education				
	No [0]	65 (90.3)	124 (89.9)	57 (85.1)
	Yes [1]	7 (9.7)	14 (10.1)	10 (14.9)
School type				
	Public [1]	68 (94.4)	135 (97.8)	67 (100)
	Private [2]	4 (5.6)	3 (2.2)	0 (0.0)
School year taught				
	1st year [1]	17 (23.6)	19 (13.8)	13 (19.4)
	2nd year [2]	17 (23.6)	29 (21.0)	4 (6.0)
	3rd year [3]	10 (13.9)	30 (21.7)	10 (14.9)
	4th year [4]	9 (12.5)	27 (19.6)	21 (31.3)
	More than one year [5]	19 (26.4)	33 (23.9)	19 (28.4)
Self-reported familiarity with digital technologies				
	No [0]	5 (6.9)	7 (5.1)	0 (0.0)
	Yes [1]	67 (93.1)	131 (94.9)	67 (100)
Training in digital technologies				
	No [0]	23 (31.9)	37 (26.8)	17 (25.4)
	Yes [1]	49 (68.1)	101 (73.2)	50 (74.6)

Note: Results are presented as n (%). Number in brackets for sex, geographical area, education, specialization in special needs education, school type, school years taught, familiarity with digital technologies, and training in digital technologies represent how each variable was dummy-coded for hierarchical regression analyses (see Table 4 and corresponding text).

**Table 3:** Descriptive and correlation values between measures (n = 290).

	1	2	3	4	5
1. Self-efficacy					
2. Positive perception	0.20 ***				
3. Negative perception	−0.42 ***	−0.32 ***			
4. Use of digital technologies overall	0.32 ***	0.31 ***	−0.28 ***		
5. Use of digital technologies for reading and writing	0.30 ***	0.29 ***	−0.25 ***	0.68 ***	
*M*	37.08	13.91	10.97	21.19	36.52
*SD*	4.98	3.33	3.31	7.64	10.81

*** *p* ≤ 0.001.

**Table 4:** Hierarchical regression analyses predicting the frequency of use of digital technologies.

			*β*	*t*	*r* ^2^
Frequency of use of digital technologies					
	Step 1				0.01 ^ns^
		Sex	0.04	0.68 ^ns^	
		Age	0.22	1.70 ^ns^	
		Geographical area	0.02	0.33 ^ns^	
	Step 2				0.04 ^ns^
	Education		−0.06	−0.95 ^ns^	
		Specialization in special needs education	0.02	0.36 ^ns^	
		School type	0.00	0.04 ^ns^	
		School years taught	−0.02	−0.42 ^ns^	
		Years of service	−0.07	−0.59 ^ns^	
		Special needs measures adopted	0.10	1.79 ^ns^	
	Step 3				0.06 ^ns^
		Self-reported familiarity with digital technologies	−0.01	−0.21 ^ns^	
		Training in digital technologies	0.05	0.88 ^ns^	
	Step 4				0.23 ***
	Self-efficacy		0.24	3.61 ***	
		Positive perception	0.25	4.31 ***	
		Negative perception	−0.12	−1.87 ^ns^	
Frequency of use of digital technologies for reading and writing					
	Step 1				0.02 ^ns^
		Sex	0.07	1.15 ^ns^	
		Age	0.28	2.13 *	
		Geographical area	−0.05	−0.80 ^ns^	
	Step 2				0.06 ^ns^
		Education	−0.04	−0.62 ^ns^	
		Specialization in special needs education	0.01	0.25 ^ns^	
		School type	0.03	0.49 ^ns^	
		School years taught	−0.17	−3.01 **	
		Years of service	−0.13	−1.10 ^ns^	
		Special needs measures adopted	0.02	0.33 ^ns^	
	Step 3				0.08 *
		Self-reported familiarity with digital technologies	−0.01	−0.17 ^ns^	
		Training in digital technologies	0.06	0.94 ^ns^	
	Step 4				0.21 ***
		Self-efficacy	0.23	3.32 ***	
		Positive perception	0.21	3.48 ***	
		Negative perception	−0.09	−1.40 ^ns^	

Note that *β* and t values are presented for variables entered simultaneously (i.e., Steps 1, 2, 3, and 4). *r*^2^ values are about the entering of each new set of independent variables in each step. Sex, geographical area, education, specialization in special needs education, school type, school years taught, familiarity with digital technologies, and training in digital technologies were dummy-coded; please see Table 1. Special needs measures were dummy-coded as 1 = only universal measures, 2 = two types of measures, and 3 = all types of measures. *** *p* ≤ 0.001, ** *p* < 0.01, * *p* < 0.05, ^ns^ *p* > 0.05.

**Table 5:** Teachers report how often they use digital technologies with different reading and writing skills.

		Never	1–2 Times per Semester	1–2 Times per Month	Every Week	Several Times a Week
Student’s skills						
	Phoneme–grapheme knowledge	58 (20.0)	22 (7.6)	25 (8.69	124 (42.8)	61 (21.0)
	Phonological awareness	47 (16.2)	30 (10.3)	32 (11.0)	126 (43.4)	55 (19.0)
	Decoding	49 (16.9)	18 (6.2)	43 (14.8)	126 (43.4)	54 (18.6)
	Reading fluency	30 (10.3)	19 86.6)	51 (17.6)	138 (47.6)	52 (17.9)
	Reading comprehension	18 (6.2)	15 (5.2)	44 (15.2)	145 (50.0)	68 (23.4)
	Vocabulary	9 (3.1)	12 (4.1)	44 (15.2)	150 (51.7)	75 8(25.9)
	Grammar	11 (3.8)	11 (3.8)	43 (14.8)	154 (53.1)	71 (24.5)
	Spelling	21 (7.2)	16 (5.5)	62 (21.4)	135 (46.6)	56 (19.3)
	Formulate thoughts or opinions	52 (17.9)	31 (10.7)	65 (22.4)	116 (40.0)	26 (9.0)
	Edit texts	36 (12.4)	27 (9.3)	78 (26.9)	120 (41.4)	29 (10.0)
	Look for information on the internet	8 (2.8)	22 (7.6)	42 (14.5)	133 (45.9)	86 (29.3)
	Present information	12 (4.1)	22 (7.6)	47 (16.2)	111 (38.3)	98 (33.8)
	Listen to active stories	14 (4.8)	16 (5.5)	70 (24.1)	129 (44.5)	61 (21.0)
	Listen to facts	16 (5.5)	20 (6.9)	80 (27.6)	123 (42.4)	51 (17.6)

Note: Results are presented as n (%).

## Data Availability

The raw data supporting the conclusions of the present article will be made available by the authors upon reasonable request.
